# Environmental Law and the Unsustainability of Sustainable Development: A Tale of Disenchantment and of Hope

**DOI:** 10.1007/s10978-022-09323-4

**Published:** 2022-09-08

**Authors:** Louis J. Kotzé, Sam Adelman

**Affiliations:** 1 Faculty of Law, North-West University, 11 Hoffman Street, Potchefstroom, 2521 South Africa; 2 Law School, University of Lincoln, Lincoln, UK; 3 Institute for Advanced Sustainability Studies, Potsdam, Germany; 4 School of Law, University of Warwick, Coventry, CV4 7AL UK

**Keywords:** Anthropocene, *Buen vivir*, Developmentalism, Ecological sustainability, Environmental governance, International environmental law, Neoliberalism, Sustainable development, Sustainable Development Goals

## Abstract

In this article we argue that sustainable development is not a socio-ecologically friendly principle. The principle, which is deeply embedded in environmental law, policymaking and governance, drives environmentally destructive neoliberal economic growth that exploits and degrades the vulnerable living order. Despite seemingly well-meaning intentions behind the emergence of sustainable development, it almost invariably facilitates exploitative economic development activities that exacerbate systemic inequalities and injustices without noticeably protecting all life forms in the Anthropocene. We conclude the article by examining an attempt to construct alternatives to sustainable development through the indigenous onto-epistemology of *buen vivir*. While no panacea, *buen vivir* is a worldview that offers the potential to critically rethink how environmental law could re-orientate away from its ‘centered’, gendered and anthropocentric, neoliberal sustainable development ontology, to a radically different ontology that embraces ecologically sustainable ways of seeing, being, knowing and caring.

## Introduction

Sustainable development is central to the current global development vision (Guruswamy 2010). It also acts both as a pivotal point of orientation of environmental law and policy, and as a *Grundnorm* that anchors and orientates global environmental governance (Kim and Bosselmann 2013). Sustainable development emerged in the 1970s with the noble idea that individuals would favour development that enhances human wellbeing and social justice while protecting the environment. It was originally conceived as a ‘discourse of resistance, fusing radical environmental consciousness with a critical rethinking of a failed development enterprise’ by highlighting the ‘scarcity and limits, affluence and poverty, global inequality, and the environmental viability of westernization’ (Carruthers 2001, p. 93).

Over the years, however, sustainable development has metamorphosed into a perverse ideological paradigm that provides the overarching ‘basis for organising the beliefs, subjectivities and values of individuals, and thus producing and reproducing a certain social order in its multiple dimensions, from the individual to the institutional’ (Gudynas 2013, p. 28). Neoliberalism acts as the broader context for the continuing hegemony of mainstream models of development, of which sustainable development is the latest and dominant incarnation (Carruthers 2001). More recently, the confluence of the Covid-19 pandemic and climate change has yet again exposed the limitations of sustainable development and the inequalities occasioned by its neoliberal mindset (Barbier and Burgess 2020; Horn 2021). These predatory mainstream development models contribute to the socio-ecological crisis of the Anthropocene by exacerbating systemic inequalities and injustices, repeatedly generating economic crises and driving habitat destruction that intensify the vulnerability of the living order (Adelman 2021a). This is anything but *sustainable* development.

This article outlines our deep concern about the perverse consequences of the central role of sustainable development as the dominant worldview that steers the current course of ‘development’, especially insofar as it manifests as a core environmental law and governance principle in the context of the Anthropocene.^1^ We concur with the view that ‘sustainable development is an ecopolitical project which might be neither sustainable nor developmental … [I]t is a palatable approach to “green-wrap” the economic and political project of “sustainable degradation” already now fully in play’ (Luke 2008, p. 1813). We argue that sustainable development, an ‘improbable idea [that] is too rarely questioned’ (Nebbia 2012, p. 101), promises what it cannot deliver due to its central contradiction between economic growth and ecological sustainability. We will further show that sustainable development is based on the false promise that endless growth is actually possible on a finite planet where the human footprint is already far greater than Earth’s ability to sustain life.^2^ This false promise cultivates, what Gudynas (2013) describes as the delusion of infinite natural assets that the capitalist system has at its disposal to expand forever, while in fact, the ambition of sustainable development is detached from the reality of ever-deepening socio-ecological destruction of a finite planet. Another concern is that the concept is so conveniently malleable that it enables its proponents to mask the negative socio-ecological impacts of relentless growth-driven development promoted by states and corporations. We are particularly concerned that environmental law is complicit in facilitating all of the foregoing to the extent that sustainable development is a *legally sanctioned* ideological palliative at the centre of environmental law and governance that enables its proponents to rationalize continuing destruction of the fragile earth system, rather than safeguarding planetary integrity (Kotzé et al. 2022, forthcoming; Richardson 2011, p. 31). Our focus in this paper is on environmental law, but the legal and regulatory responses adopted by the international community in response to environmental degradation have implications for the broader substantive and systemic principles and processes of law.

In the following section, we offer a brief contextual overview and critique of growth-driven development and the associated ideology of developmentalism and neoliberal globalization that underpins sustainable development. We endeavour to reveal the many contradictions at the heart of sustainable development’s history and its different iterations by global institutions and associated legal and political apparatus. We then provide a deeper critique of the role of sustainable development in environmental law and governance to demonstrate how it perpetuates epistemologies of mastery over a vulnerable living order at a time when we urgently need epistemologies of humility and care as alternatives to mainstream models of development. The aim in this part of the discussion is to show how environmental law has played a crucial role in legitimising a wide range of socio-ecologically destructive practices around the world in the name of sustainable development. The article concludes more hopefully with an examination of an attempt to reimagine alternatives to the sustainable development paradigm in the form of the indigenous onto-epistemology of *buen vivir* that articulates an ethics of humility and care alien to sustainable development. While *buen vivir* has not yet managed to fully replace sustainable development in the countries where it operates (at least not in practice), the influence of this worldview is already evident in some laws and policies. This suggests that its acceptance in environmental law and governance as an alternative to sustainable development is more feasible than might be expected, and that there is hope for pursuing a radically different future legal trajectory of planetary care that focuses on the well-being of the entire living order.

## The Problems with Development, Developmentalism and Growth

Development, the fulcrum on which the notion of sustainable development revolves, emerged after the Second World War as a way of incorporating the erstwhile Third World into the global economy on unequal terms (Escobar 2011). As Hettne writes, development is ‘one of the oldest and most powerful of all Western ideas’ (1995, p. 29). Development was promoted as the means whereby postcolonial states:

could achieve economic growth, reduce poverty and promote social justice. In principle, development is a process of social change designed to improve the wellbeing of people. In practice, it has regularly manifested itself as underdevelopment or maldevelopment so that its scope and rationale have been vigorously contested. (Adelman and Paliwala 2021, p. 1)

The idea of development was promoted by the West as the means to save benighted and feckless developing countries from themselves. The concept attracted criticism from the outset but its advocates defended it as a form of progress through modernisation, extractive industrialisation, and economic growth as the primary means of promoting human wellbeing and as the sole expression of material progress (Gudynas 2013). Supporters of development conveniently discount the inconvenient truth that all economic activity is intrinsically destructive when it involves the exploitation of the foundations of life on Earth (Luke 2008). Mainstream, hegemonic models of development have been particularly heedless of environmental destruction despite repeated warnings about the limits to growth, increasing evidence of earth system decay, fast approaching planetary boundaries, and a looming sixth mass extinction event (Barnosky et al. 2011). Profit has persistently trumped people and the planet, driven by the logic of capital accumulation as the only measure of progress and well-being. One effect is that the tenacity of such mainstream models of development has successfully crowded out and marginalized alternative visions of progress and well-being in their pursuit of ‘uninterrupted growth towards a civilisation in the image and likeness of the countries of the North’ (Alcoreza 2013, p. 147).

Today it is almost tautological to argue that development requires growth. Despite attempts by Sen (1999) and others to construe development as freedom through the capabilities approach—which gave rise to measures of human-centred development such as the Human Development Index—the concept of development remains irredeemably anthropocentric, economistic, extractivist, and growth-driven (UNDP n.d.). By the 1960s, development had become an ideological end in itself in the form of *developmentalism*;

an ideological orientation characterized by the fetishization of development, or the attribution to development of the power of a natural (or even, divine) force which humans can resist or question only at the risk of being condemned to stagnation and poverty. The ideology renders opaque the historical forces that have shaped the idea of development. It also disguises the social and political forces that have played, and continue to play, a crucial part in endowing it with the power to dominate human consciousness. (Dirlik 2014, pp. 30–31)

The intrinsic flaws of developmentalism, and by extension of neoliberal sustainable development, are only now belatedly acknowledged (at least in part) by institutions such as the United Nations Environment Programme (UNEP), a longstanding promoter of green capitalism and the green economy (Carvalho 2001). UNEP’s position aligns with the mainstream proposition that sees green capitalism and sustainable development as a combination of ‘environmental and sustainability discourses with industrial and economic policy ones, in search of “win–win” solutions and virtuous cycles of progress and prosperity’ (Bina 2013, p. 1024). It has taken several years for the world’s foremost intergovernmental environmental protection champion to finally accept that ‘the current mode of development degrades the Earth’s finite capacity to sustain *human well-being*’ (UNEP 2021, p. 13, our emphasis). But having linked itself so tightly to sustainable development, UNEP is trapped within the mindset of human focused developmentalism that consistently sidelines ecological sustainability. This is also evident in its acknowledgment that ‘environmental changes are undermining hard-won development gains … [and] are impeding progress towards ending poverty and hunger, reducing inequalities and promoting *sustainable economic growth*’ (UNEP 2021, p. 13, our emphasis). Global governance institutions responsible for environmental protection are clearly mired in a discursive straitjacket that makes it difficult or even illegitimate for them to question sustainable development orthodoxy. The nub of the problem is that all mainstream models of development are predicated upon economic growth which flows from the dogmatic insistence that the current mode of purportedly *sustainable* development can be tweaked in the right direction. This makes it impossible to reconcile ecological sustainability and sustainable economic growth. Given earth system limits, endless growth cannot be sustainable; after all, humanity currently uses 74 per cent more than the planet’s ecosystems are able to regenerate—or 1.7 Earths,^3^ while four of the nine planetary boundaries have already been crossed, and the others are fast approaching (Rockström et al. 2009). Yet, ‘growthism’ (Hickel 2020, p. 89) remains the main driver of neoliberal globalisation and its devastating effects on our shared world (Fraser 2021; Springer 2016), while proponents of green capitalism such as UNEP continue to maintain that it is possible to grow our way to sustainability, among others, through decoupling; the idea—or fantasy—that economies can grow without increasing environmental pressures (Fletcher and Rammelt 2017).

It seems that the classical Western idea of growth-driven, extractive development has been regularly declared dead but persists in a zombie-like form (Gudynas 2011). The reality is that the root of the socio-ecological crisis lies, as Nebbia (2012, p. 97) says, in the ‘myth of economic “growth” and the endless increase of its only form of measurement’: gross domestic product (GDP). GDP remains the predominant measure of development, growth and national virility, despite persistent and growing criticism (Daly 2013; Higgs 2014). It is primarily a measure of market transactions and output that ignores social costs, inequality, and environmental destruction (Hickel 2020). It has long been clear that ‘GDP [is] … a poor proxy for societal wellbeing, something it was never designed to measure’ (Ward et al. 2016, pp. 11–12). This gives rise to the paradox that the fetishization of economic growth is counterproductive because an increasing proportion of GDP is comprised by defensive or negative expenditure, such as the costs of adaptation and pollution (Stiglitz et al. 2020). In developed countries, growth has effectively become *un*economic because its benefits no longer exceed its costs (Kallis 2018). Accordingly, there are increasing calls to ‘reject the temptation, often unconscious, to accept gross domestic product as an objective measure of social well-being and economic progress’ (England 1998, p. 102); and to adopt instead alternative approaches, such as Bhutan’s ‘gross national happiness index’, as a measure of progress (Brooks 2013).

Some proponents of development acknowledge the limitations of growth and GDP but nevertheless seem unable to abandon these measures of neoliberal progress, wealth, and well-being. For example, an assessment commissioned by the United Kingdom Treasury titled *The Economics of Biodiversity: The Dasgupta Review* concedes that ‘economic progress should be read as growth in inclusive wealth, not growth in GDP nor growth in any of the other ad hoc measures that have been proposed in recent years such as the UN’s Human Development Index’ (Dasgupta 2021, p. 121). Yet, in the same breath, the review perpetuates the contradiction that ‘GDP growth is, in principle, compatible with sustainable development … [and] per se is not an obstacle to sustainable development’ (Dasgupta 2021, pp. 335–336). The review defines sustainable development as a balance between humanity’s global impact on the biosphere and the biosphere’s regenerative rate (Dasgupta 2021, p. 33) and accepts that nature has intrinsic value, but seems unable to break free from the neoliberal mindset that views nature as a reservoir of capital and services that must be priced in order to be valued and protected. The review’s insistence that ‘ecosystems are capital goods’ (Dasgupta 2021, p. 52) indicates that it is merely a more sophisticated way of costing the Earth and its ‘resources’.

In sum, sustainable development is ‘increasingly regarded either as internally self-contradictory (an oxymoron) or, at best, plagued by ambiguous or distorted definitions’ (Johnston et al. 2007, p. 60). It is part of a terminology that some justifiably regard as thoroughly degraded: ‘words like green, sustainable, “net-zero”, “environmentally friendly”, “organic”, “climate-neutral” and “fossil-free” are today so misused and watered down that they have pretty much lost all their meaning’ (Rowlatt 2020). Sustainable development has made a substantial contribution to this degradation as it has morphed into a ‘highly questionable and manifestly unsustainable’ self-serving paradigm, ‘whereby economic growth and exploitation economics act as primary drivers of resource management’ (Johnston et al. 2007, p. 61). The foregoing analysis suggests that the core problem with sustainable development is that it is merely another form *of* development rather than an alternative *to* mainstream development. Whereas the former envisages development in a different form, the latter regards the idea of development as irredeemably unsustainable, notably because dominant ideas of progress and well-being remain trapped within the stifling, interlinked confines of economic growth, GDP, and developmentalism. Sustainable development as we know it today is therefore, in fact, unsustainable. It is a paradigm that encourages the ‘deification of the market, private property, and the conquest of goods’; which leads to the gradually intensifying destruction ‘of any ideal of a different relationship between human beings, objects, and natural resources’ (Nebbia 2012, p. 102).

In the following section we interrogate how environmental law plays a key role in further cementing sustainable development as its cornerstone principle, and as the guiding mantra of a neoliberal world order where growth without limits is legitimised and even encouraged. The irony is that environmental law is the body of law primarily responsible for protecting a vulnerable living order against the sort of socio-ecological destruction occasioned by the principle of sustainable development that environmental law fully embraces.

## (Un)sustainable Development and Environmental Law

The problem of intrinsically unsustainable development is one part of the problem; equally problematic are the conflicting assumptions of sustainable development that are hardwired into environmental law, and that drive socio-ecological destruction. The fateful link between development and environmental protection in environmental law was made when neoliberal globalisation became a hegemonic ideology. As a result, during the last five decades, environmental law—particularly international environmental law—has been instrumental in entrenching sustainable development as another, ostensibly better form *of* development (Lang 1995). As we will show below, the perverse result of sustainable development’s embeddedness in law and governance institutions is that it has weakened their capacity to ensure planetary integrity and the collective wellbeing of the living order: ‘under the paradigm of sustainable development, current international law has been unable to shape a real or equitable answer to the global ecological crisis’ (Manzano et al. 2016, p. 382).

International environmental law has played a pivotal role in turning sustainable development into a normatively, politically, economically, and socially powerful concept, and is therefore complicit in promoting a socio-ecologically destructive understanding of sustainable development in a body of law that is supposed to be primarily concerned with ensuring planetary integrity (Kotzé 2019a). International environmental law thereby legitimises a wide range of socio-ecologically destructive practices under the banner of sustainable development (Heydon 2019). In fact, international environmental law and sustainable development have become so deeply interwoven that some scholars claim to discern the existence of a new body of law called ‘international sustainable development law’ (Cordonier-Segger 2004). Whether or not one agrees with this taxonomy, sustainable development appears to have become the foundational ‘constitutional’ principle of international environmental law (analogous to Hans Kelsen’s notion of a *Grundnorm*) (Guruswamy 2010)—possibly its *raison d’etre* and, of greater concern, its core ethical orientation. This is reflected in the view that ‘the concept of “sustainable development” has entered the corpus of customary international law’ (Sands et al. 2018, p. 219). This is significant because customary international law has been characterised as a form of international constitutional law (De Wet 2006), which, by implication, affords sustainable development global ‘constitutional’ status insofar as a global constitution does exist in the realm of international law (Kim and Bosselmann 2013; Kotzé 2015).

As sustainable development becomes more deeply woven into hegemonic neoliberal discourses and gains wider acceptance—for example, in the Sustainable Development Goals (SDGs)—decision-makers are increasingly caught on the horns of a dilemma: knowing that state practice has turned sustainable development into Hobson’s choice, it is pursued despite, and often precisely because of, its intrinsic oxymoronic flaws, which facilitates the unambitious path dependency of environmental law. The body of norms designed to promote global environmental governance is usually created through formal consensus. The problem with the requirement for consensus is that it obscures the imbalanced diplomatic, economic, political and ideological power of states that produce agreements that coalesce around lowest common denominators rather than ambitions based upon the best available science (Caballero 2019). The Paris Climate Agreement, and all its successive Conferences of the Parties (COPs), including the most recent COP26 in Glasgow, is an example (Masood and Tollefson 2021).

This trend has characterised the history of international environmental law. It is not coincidental that sustainable development and neoliberalism emerged at roughly the same time and became increasingly tightly linked following the World Conference on Environment and Development in 1987 with the publication of the World Commission on Environment and Development’s (WCED) Brundtland report; a publication that has been described as a veiled attempt to ‘appease the [pro-growth] economic establishment’ at the time (Nebbia 2012, p. 101). Consensus was achieved at WCED because the report’s emphasis on development mollified developing countries while its focus on economic growth assuaged Western concerns (Borowy 2013). Without this compromise, the concept would not have emerged in the Brundtland formulation. The report formed the basis for an international consensus precisely because it suppressed the contradiction between the ideal of endless extractive growth on the one hand, and the real and sobering limits of the earth system on the other hand; and it offered the delusion that mainstream models of development could easily be tweaked to achieve social justice for everyone everywhere (Lafferty 1996). The Brundtland Report made it clear that ‘what is needed now is a new era of economic growth—growth that is forceful and at the same time socially and environmentally sustainable’ (WCED 1987, p. xii). In retrospect, the Brundtland Commission’s mandate was to produce a neoliberal template for green capitalism (Higgs 2014). This inevitably perpetuated the inescapable logic that ‘by their own intrinsic laws, capitalist societies can survive only through a continuous growth in the production and consumption of goods [which] occurs at the cost of a growing extraction and contamination of the planet’s natural resources’ (Nebbia 2012, p. 101).

The Brundtland Report was facilitated by the linkage between sustainable development and environmental protection that had been tentatively drawn at the UN Conference on the Human Environment in 1972—a linkage that became progressively more explicit at successive international environmental conferences. The Stockholm Conference provided the anthropocentric, utilitarian and gendered^4^ context for ensuing environmental negotiations, and the creation of international environmental norms that prioritise the needs of certain humans over those of other vulnerable species and the biosphere. The preamble to the Stockholm Declaration, for example, states: ‘both aspects of man’s environment, the natural and the man-made, are essential to his well-being and to the enjoyment of basic human rights’.

During the 1992 Earth Summit, states sought to reconcile anthropocentric sustainable development with the need for biocentric and ecocentric measures to protect the biosphere, but made little progress. This is evident in the first principle of the Rio Declaration, which states: ‘human beings are at the centre of concerns for sustainable development’. Article 1 of the Convention on Biological Diversity, a key agreement flowing from this conference, calls for the conservation of biological diversity and the sustainable use of its components, but the underlying ethos of this convention is that nature exists for, and can be *sustainably developed* to meet human needs and interests (Somsen and Trouwborst 2021).

In 2000, ‘environmental sustainability’ was politically but inadequately institutionalised in Goal 7 of the Millennium Development Goals (MDGs) (Fehling et al. 2013). The aim of Goal 7 was to ‘Ensure Environmental Sustainability’ and one of its targets was to: ‘integrate the principles of sustainable development into country policies and programmes and reverse the loss of environmental resources’. MDG 7 unfortunately veers between ‘strong’ environmental sustainability in its title and the ‘weaker’ form of sustainable development in its targets.

The SDGs replaced the MDGs in 2015 (UN 2015). Like the MDGs, the SDGs are not legally binding, but have some normative power and steering effects through their softer ‘governance through goals’ approach (Biermann et al. 2017), and insofar as the SDGs are linked to the more deliberate steering effects of international (environmental) law (French and Kotzé 2018). The absence of an overarching environmental or ‘planetary’ goal in the SDGs is remarkable (Brandi 2015) with environmental protection consigned to Goals 13, 14 and 15 way down at the bottom of the list of 17 goals. ‘[N]umerically and rhetorically, the list effectively makes development goals more important than the environmental goals’ (Craig and Ruhl 2019, p. 1). Eisenmenger et al. argue that ‘the SDGs fail to monitor absolute trends in resource use and thus prioritize economic growth over ecological integrity’; they ‘rely mainly on those institutions responsible for unsustainable resource use, and partly propose measures that even reinforce current trends towards less sustainability’ (2020, p. 1101). Others have demonstrated the extent to which the SDGs reinforce the anthropocentric, neoliberal models of development on which international environmental law has been constructed over the past fifty years (Kotzé 2018). One reason for this is that the SDGs ‘adopted a technocratic approach which tried to pursue both economic growth and environment, highlighting measures such as “decoupling”, “resource efficiency” and “integrated management” as key solutions’ (Elder and Olsen 2019, p. 71)—an approach that is deeply embedded in neoliberal dogma. The result is that techno-managerial green capitalist ‘solutions’ have been locked into global environmental law and governance responses, including the SDGs, on the flawed assumption that free markets can undo environmental destruction.^5^ Other examples are the ‘green economy’ or payment for ecosystem services, while a more recent incarnation of techno-managerial green capitalist solutions is the attempt to ‘green’ capitalism through green or blue impact bonds. As DePuy et al. say, all of these neoliberal market based approaches ‘are grounded in the reduction, abstraction, and commodification of the natural world, the “cosmology of late capitalism”, and ultimately “the creation and production of disembedded, pacified things”’ (2022, p. 5).

The fact that the SDGs appear to reflect cross-cultural agreement says more about global power structures and hegemonic ideas of development than it does about the effectiveness of measures for achieving global ecological sustainability. On face value, the 17 goals and 169 targets in the 2030 Agenda constitute an attempt to concretise the meaning of sustainable development. Goals such as ending poverty and hunger, promoting health and wellbeing, achieving equality, protecting carbon sinks, and dealing with climate change are important, but the 2030 Agenda provides little guidance about how they should be achieved in an inclusive way (Gupta and Vegelin 2016). For example, Goal 8, to ‘promote *sustained*, inclusive and *sustainable* economic growth’ (our emphasis) is fundamentally contradictory because sustained economic growth cannot logically be sustainable if it depends on the limited foundations of life on Earth, as it does. As Washington argues, in a ‘finite world, we need to accept once and for all that sustainability *cannot* be about further growth. This challenge remains critical, though still denied’ (2015, p. 36, emphasis in original). The 2030 Agenda contains lofty ambitions and stirring rhetoric, but no reference to or measure of strong ecological sustainability, or the need to protect planetary integrity, and no resources to enable the world, especially the global South, to implement the SDGs in genuinely sustainable ways that promote inclusive well-being (Kotzé et al. 2022, forthcoming). Tellingly, the 2030 Agenda contains more references to debt sustainability than ecological sustainability (Adelman 2018). In the SDGs, as in much international environmental law, truly *sustainable* development therefore appears as a mantra that can be dispensed with as and when economic and political expediency dictates.

The risk of continuing down this well-trodden path is that environmental law and governance plays a key role in reducing sustainable development to alluring promises that cannot be achieved while it inhibits effective responses to multiple complex, multi-scalar and interlinked existential socio-ecological crises in ways that are fair, just and inclusive (to ‘leave no one behind’—the central promise in the 2030 Agenda). It is rather the case that the combination of sustainable development and neoliberal globalisation has remorselessly increased inequality within and between states and made poverty, food, water and energy insecurity endemic (Lonergan 2000). Environmental law is unable to tackle this challenge because its pursuit of sustainable *development* merely perpetuates the patterns of systemic inequality and insecurity occasioned by the very principle it deifies.

At WCED, the Brundtland Commission argued that sustainable development offered solutions to multiple crises: ‘an environmental crisis, a development crisis, an energy crisis. They are all one’ (WCED 1987, p. 4). Since then, these crises have deepened and become entwined with other crises such as the 2008/2009 global financial crisis, the Covid 19 pandemic, and the climate crisis. These overlapping crises have brought us to the brink of catastrophe (Biggs et al. 2011). They also demonstrate the limitations of neoliberal understandings of vulnerability and resilience in the face of pandemics and socio-ecological breakdown, and vindicate the view that ‘to some degree or another neoliberalism has always been a creature of crisis’ (Peck 2010, p. 106). The Covid 19 pandemic, for example, highlights the connections between socio-economic class, ethnicity and race, precarity, and vulnerability to infection. The structural inequalities of the global economy—reflected in the unequal production and distribution of vaccines—reveal the emptiness of the high-blown rhetoric about a global partnership for sustainable development in the 2030 Agenda (French and Kotzé 2022, forthcoming). These structural inequalities place a large question mark against the aim of Goal 17, to strengthen ‘the means of implementation and revitalize the Global Partnership for Sustainable Development’—a partnership that is difficult to discern in the lack of solidarity in the international response to Covid-19.

A critical concern is that calls to ‘build back better’^6^ in a socio-ecologically just way when the pandemic is brought under control will be drowned out by the desire to return to ‘normality’, as if what was regarded as ‘normal’ prior to the pandemic was not profoundly abnormal. If returning to normality means business as usual, the pandemic will be a wasted opportunity rather than a generational inflexion point. To the extent that the pandemic demonstrates one of the consequences of socio-ecological destruction and human interference in biological lifeworlds, it signals the urgent need for a different trajectory for the pursuit of the wellbeing of the entire living order. While environmental law will have to play an important role in pursuit of such a radically different trajectory, the history of sustainable development, within and outside the domain of environmental law, suggests that this principle cannot be part of such a radically different trajectory.

## An Alternative for the Wellbeing of a Vulnerable Living Order

Environmental law will have to discard sustainable development as its central orientation, and must pursue alternative worldviews that can counter sustainable development’s tenaciously malign influence on the world. Central to any effort of environmental law’s relinquishment of sustainable development will have to be the acknowledgement that merely altering environmental law’s content—by incorporating new principles such as *in dubio pro natura*,^7^ adopting provisions to declare ecocide an international crime,^8^ or recognising a global right to a healthy environment,^9^ for example—is necessary but insufficient to deal with the exigencies of the Anthropocene as long as deep-seated problems perdure with environmental law’s *form*. Deeper change will be needed to re-orientate environmental law away from neoliberal sustainable development, to alternative, ecologically sustainable ways of seeing, being, knowing and caring.

Any reorientation effort will have to consciously embrace a critical awareness of the deeper and enduring structural legacies of Enlightenment rationality such as anthropocentrism, binary thinking, and the imbalance between legal protection of property and nature that are all being perpetuated by sustainable development. Liberal law, after all, has been deformed by rational thinking that places *Anthropos* at the centre of an environment where ‘nature’ merely acts as a backdrop for and sustenance of the many hierarchies that liberal law creates and perpetuates between living beings (Grear 2015). In addition to its tendency to selectively privilege human sovereignty, international environmental law’s rationalist centric orientation is also evident in its geography; its territorialised sovereign-centric rationality that is at odds with the transboundary nature of global environmental harms (Kotzé 2019a). Sustainable development operates within this rationalist Enlightenment context of liberal law that creates a toxic mix which enables an emboldened anthropocentric, top-down, techno-managerial framing of socio-ecological breakdown, where the hu*man*, stands at the centre of all concerns (Kotzé 2019b). Such a framing reproduces hierarchy while ignoring the insights of alternative epistemologies about relationality and the vulnerable relationships between humans and the non-human world (e.g. Deckha 2013). Sustainable development embodies the circumscribed, hidebound, traditional and centric legal thinking that has led a growing number of scholars to seek ways to reimagine law and legal theory in holistic ways that address hierarchies of exclusion—of marginalized humans, the unborn, and non-humans (e.g. Burdon 2011; Grear et al. 2021).

The problem of centric thinking, and particularly the extent to which such thinking privileges the interests of some humans, leads Philippopoulos-Mihalopoulos (2011, p. 5) to call for a ‘*radical theoretical reconfiguration of environmental law*’ (emphasis in original); leading to a critical environmental law that ‘exerts a radical critique of traditional legal and ecological foundations, while proposing in their stead a new, mobile, material and acentric environmental legal approach’ (Philippopoulos-Mihalopoulos 2015, p. 57). Such a reconfiguration leads to an open ecology where the human is decentered from ‘problem spaces’, where it is possible to reveal the role and place of human-non-human interconnections, while creating a space for the enactment of new ontological categories (DePuy et al. 2022). This is necessary to guide our ‘world-views; conceptions about people and the way they interact; ethical frameworks and values; assumptions about what exists and what does not exist; and the paths to knowledge and objectivity’ (Villalba 2013, p. 1430).

We already observe the emergence of alternative, ecologically sustainable ways of seeing, being, knowing and caring that offer opportunities to appreciate how ‘worlds are known and enacted, so as to more ethically and effectively navigate contemporary socioecological challenges facing the planet and the human-nonhuman relations upon which its health depends’, while at once enabling a critical interrogation of ‘questions of power and the ways that dominant discourses, practices, and institutions [including sustainable development] shape the worlds in which people live’ (DePuy et al. 2022, pp. 3–4). These alternatives can replace, and are already gradually replacing in some legal systems, the hubristic epistemologies of dominance and mastery that have brought us to the precipice.

In this final section we briefly highlight such an example of an indigenous onto-epistemology that could offer an alternative to sustainable development, namely, *buen vivir* (living well). Space precludes a full discussion of this worldview and of its rich epistemological, economic and political implications. But there seems to be some agreement on its potential, at least conceptually, to influence environmental law and governance in the longer term by providing an opportunity to dissolve modernist dualisms, and to promote more expansive ways of seeing, being, caring and knowing that can redefine sociality and relationality in a decentered, all-inclusive and non-hierarchical ‘ecological’ space (e.g. DePuy et al. 2022). We believe that *buen vivir* can guide, to some extent, the type of radical reconfiguration of (‘Western’) environmental law that is being called for^10^; if not fully in practice (yet), then at least in theory.

*Buen vivir* is a central idea in Andean cosmovisions. It promotes an alternative to neoliberal sustainable development; a concept alien to Andean cosmovisions, conceptual categories, and languages of indigenous communities (Walsh 2010). In seeking to bridge the abyssal divide between indigenous, pluriversal thinking and the universalistic pretensions of liberal law and governance (Escobar 2018), *buen vivir* promotes a biocentric counterweight to anthropocentrism in which *Pachamama* (Mother Earth) is understood as an ever-present deity who is the source and sustainer of all life, of which humans are only a small part. *Buen vivir* offers alternative forms of law and governance to protect *Pachamama* that reject the Cartesian society-nature dualism. Gudynas writes that *buen vivir* ‘moves away from the prevalence of instrumental and manipulative rationality. It rejects the modern stance that almost everything should be dominated and controlled, including people and nature, so that they become means to exploitative ends’ (2011, p. 445). In this sense, *buen vivir* ‘assumes a relationship of belonging rather than domination or exploitation’ (Caria and Domínguez 2016, p. 20). *Buen vivir* also eschews the notion that human beings are at the centre of all concern and the only source of values, and it shuns modernity’s obsessions with growth and progress because it does not conceive a beginning or end in time in the way that visions of progress and development from point a to b (or from poor to rich) do in the context of European modernity. This means that ‘there can be no “development” insofar as there is no preliminary situation of underdevelopment’ (Villalba 2013, p. 1430).

In Andean cosmovisions, well-being is possible only within a community understood in an expansive sense that also includes non-humans. Compared to GDP—sustainable development’s key measure of well-being—*buen vivir* instead involves a broader, more inclusive notion of well-being and cohabitation with the non-human world, which it views as an essential, constitutive element of social life with intrinsic value (Gudynas 2011). Well-being flows from communal life in harmony with nature and it is consistent with principles of reciprocity, complementarity, and relationality. In this sense, well-being is related to a ‘life in fullness’, which means ‘a life of material and spiritual excellence expressed harmoniously and in relation to all beings, as well as a community’s internal and external equilibrium’ (Villalba 2013, p. 1430); it is an axiological principle geared towards the generation of values of emancipation (Alcoreza 2013). As a subaltern conception of well-being (Van Norren 2020)*, buen vivir* derives from a rich body of indigenous and local knowledge that have been shown to promote ecological sustainability (IPBES 2019), as well as alternatives to sustainable development (Watene and Yap 2015).

*Buen vivir* already exercises a growing influence on Latin American jurisprudence through its incorporation in the national development plans, and constitutional and statutory provisions in Bolivia, Ecuador and Colombia (Kotzé and Villavicencio Calzadilla 2017; Villavicencio Calzadilla and Kotzé 2018), and the growing trend of giving rights to nature (Gellers 2021; O’Donnell 2018). There are also some encouraging developments that show *buen vivir* is starting to confront mainstream development models in practice. In 2011, the first court decision was handed down in the Vilcabamba River case in Ecuador, upholding that country’s constitutional rights of nature provisions.^11^ In 2017, Colombia’s Constitutional Court ruled that the Atrato River possessed rights to ‘protection, conservation, maintenance, and restoration’ and established joint guardianship arrangements shared between indigenous communities and the national government.^12^ In April 2018, the Colombian Supreme Court issued an opinion in response to a case brought by a group of young people against the government, stating that ‘for the sake of protecting this vital ecosystem for the future of the planet’, it would ‘recognize the Colombian Amazon as an entity, *subject of rights*, and beneficiary of the protection, conservation, maintenance and restoration’ that national and local governments are obligated to provide under Colombia’s Constitution.^13^ At the international level, in 2017, the Inter-American Court of Human Rights issued a landmark Advisory Opinion recognizing the right to a healthy environment as ‘fundamental to the existence of humanity’.^14^ The Opinion confirmed extraterritorial jurisdiction for transboundary environmental harms; the autonomous right to a healthy environment; and State responsibility for environmental damage within and beyond the State’s borders. Taking an ecocentric approach, the Court linked the right to a healthy environment to the rights of nature, which it identified as an emerging legal trend in Latin America.

But this alternative worldview does not come without its own inherent challenges, contradictions, disingenuities, and false promises. While optimistic about its potential, we are aware that the implementation of *buen vivir* in Latin America has been contradictory and uneven and that one must always guard against romanticising indigenous onto-epistemologies (Ranta, 2020). Such romanticism could rightly be seen as an effort to maintain an elusive ‘utopic horizon’ that is based on an irrational ‘pathos that flows from the inner emotional sphere rather than from a rational understanding of the world’, where the ‘fantasy of returning to an ancient (or ideal) state of fullness in which the desire itself—the enjoyment of expectation—prevails over the actual probability of fulfilling it’ (Caria and Domínguez 2016, p. 27).

The reality is that any perceived or real transformations of laws, politics, and ultimately, of societies themselves, that are brought on by alternative worldviews such as *buen vivir*, will not be immediate, unequivocal or even entirely successful. We need to recognise that *buen vivir* remains a work-in-progress, ‘rather than a constitutional declaration, it is an *opportunity* for the collective creation of a new form of organizing life itself’ (Caria and Domínguez 2016, p. 2, our emphasis). We must also be aware that ‘the expression of the principles and rights of Buen Vivir should not be confused with their implementation and attainment, for the latter requires much more time’ (Villalba 2013, p. 1437). Moreover, as we have shown earlier, the power of the neoliberal sustainable development world order is formidable, and the many deeply vested interests that sustain sustainable development will continue to resist attempts to dismantle them and undo their tenacious grip on society. Where they occur, transformations brought on by *buen vivir* will more likely be gradual ‘staged transitions’ (Villalba 2013, p. 1436) interspersed with smaller victories that are scattered over time, and political, legal and social spaces. We clearly see, for example, the many challenges posed by the realities of actually implementing *buen vivir* playing out in the so-called ‘Latin American paradox’, in terms of which unabated mining, hydrocarbon activities, and monoculture exports continue to fund social spending and public works (Lang 2013); a phenomenon that Gudynas (2013, p. 25) calls, ‘progressive neoextractivism’. This paradox points to the very real danger of *buen vivir* becoming a convenient political window dressing tool—similar to sustainable development—that disingenuously masks business-as-usual developmentalism and exploitative practices promoted by the very governments that have enshrined *buen vivir* in their laws and policies. In the case of Ecuador, for example, Caria and Domínguez show how several ‘key policies implemented in recent years reveal a deep contradiction with buen vivir principles’ (2016, p. 19). There is a very real risk that ‘the same idea of development that was circulating in the 1960s and 1970s has reappeared in new guise’ (Gudynas 2013, p. 25).

In short, while *buen vivir* evidences the rising disenchantment with mainstream models of development as we are entering a new post-neoliberal period (Villalba 2013), it is also clear that it is not a panacea. But it does reflect an aspiration to explore alternatives to sustainable development, and it hints at the possibility of dealing in alternative ways with unsustainable growth and development, explores what it means to be human in the Anthropocene, offers an alternative framing for human-non-human relations, and promotes ethics of planetary integrity (Adelman 2021b). Ultimately, *buen vivir* can serve as a discursive ‘platform’ of sorts, ‘which is arrived at from different traditions and a diversity of specific positions; where the substantive critique of development as ideology is shared and alternatives to it are explored (Gudynas 2013, p. 35). Importantly for present purposes, *buen vivir* also points to a possible different trajectory for environmental law by means of which it can address the scale and urgency of the Anthropocene’s socio-ecological crisis by relinquishing its reliance on sustainable development and becoming, in the words of Philippopoulos-Mihalopoulos ‘new’, more ‘mobile, material and acentric’ (2015, p. 57).

## Conclusion

The failure of sustainable development to address the deepening socio-ecological crisis indicates that it is fatally disconnected from Anthropocene reality. As the Chinese proverb has it, the moral of sustainable development is that we should be careful what we wish for because it may come true. Those who believe sustainable development is capable of being reinterpreted and rebranded, such as UNEP, have yet to provide a cogent explanation of how to resolve the oxymoron at its core. The impossibility of doing so underpins our contention that it is not credible to believe that sustainable development will ever be genuinely sustainable. It should therefore be rejected as irredeemably flawed for the reasons we have argued above and that we summarize below.

First, sustainable development is too deeply implicated in the myriad social and legal systems, institutions and practices that have created the unsustainable, uneven and unjust world in which we live. Perversely, its influence is inversely proportionate to its protection of a vulnerable living order. Second, sustainable development *is* business as usual; it is a conservative, if not reactionary, ideology at odds with the radical transformations urgently required to confront the Anthropocene’s rapidly unfolding and uneven socio-ecological crises. Third, in the teeth of scientific evidence, sustainable development holds out the delusion that infinite exponential growth is possible on a finite planet. Fourth, sustainable development cannot be a socio-ecologically friendly principle that sustains life on Earth unless and until it prioritises ecological sustainability and planetary integrity above growth and profit.

But sustainable development is not written in stone; it is a socio-political choice. Social and political struggles rooted in *buen vivir* demonstrate that it is feasible to reimagine alternatives that are open to the possibility of embracing a ‘relational sense of solidarity that recognizes that the subjugation and suffering of one is in fact indicative of the oppression of all’ (Springer 2016, p. 289). Abandoning sustainable development will specifically require a paradigm shift in environmental law, policy and governance. As Richardson argues, ‘better principles to guide the redesign of our environmental decrees and standards are necessary’ (2011, p. 31). As an alternative *to* development, and conscious of the many challenges associated with implementing it, *buen vivir* offers a hopeful opportunity to explore social, economic, political and legal orders that are radically different to sustainable development. The Covid-19 pandemic has shown that what was previously regarded as impossible can rapidly become possible in the face of an existential threat. In the small window of opportunity that remains, radical transformation that is driven by alternative worldviews is the only rational response to the unfolding socio-ecological crisis.

## Footnotes

1 The idea of the Anthropocene is not uncontroversial. Some caution that while it helpfully illuminates *Anthropos*’ assumed telluric force and geological power, it unfairly universalises ‘the human’ impact on the earth system (Grear 2015; Hornborg 2019). It remains the case that only a small, privileged subset of the human population is responsible for most of the harm inflicted on a vulnerable living order.

2 A recent study estimated that ‘no country meets basic needs for its citizens at a globally sustainable level of resource use … the universal achievement of more qualitative goals (for example, high life satisfaction) would require a level of resource use that is 2–6 times the sustainable level, based on current relationships’ (O’Neill et al. 2018, p. 88).

3 See Global Footprint Network (2022).

4 Elliott (1996, p. 20) shows how the concerns of women have been superficially added *on* instead of *in* to mainstream environmental law and governance debates, mainly through special conferences such as Stockholm; but even in these instances the ‘language of the environment debate is itself a gendered one, even where it pretends to be generic; and women have been silenced in this way’.

5 The Stern Review described climate change as ‘the greatest market failure in history’ (Stern 2007, p. 1). The review is the predecessor to the Dasgupta Review discussed above.

6 See, for example, the call to ‘build back better’ that was recently been made by United Nations Special Rapporteur on Extreme Poverty in an interim report titled ‘The “just transition” in the economic recovery: eradicating poverty within planetary boundaries.’ (UNGA 2020).

7 In 2017, the Global Pact for the Environment initiative sought, but ultimately failed, to introduce more ambitious principles for international environmental law such as *in dubio pro natura* (Kotzé and French 2018).

8 There has been a recent proposal to include ecocide as a crime in the Rome Statute of the International Criminal Court (ICC) (Stop Ecocide Foundation 2021). If this proposal is accepted, it would enable the prosecution of ecocide in the ICC, alongside genocide, war crimes, crimes against humanity, and the crime of aggression.

9 In October 2021, the United Nations Human Rights Council (UNHRC), for the first time recognized the right to a clean, healthy and sustainable environment as a human right (UNHRC 2021).

10 *Buen vivir* is not only relevant or applicable in an indigenous context. Villalba (2013, p. 1433) says that while *buen vivir’s* rejection of modernity could lead to the idea that *buen vivir* is only possible in indigenous contexts, ‘a broad spectrum of alternative Western thinking that is critical of development could be also understood as a search for Buen Vivir’.

11 *Wheeler v Director de la Procuradur a General del Estado en Loja*, Judgment, Provincial Court of Loja, Case No. 11121-2011-0010.

12 Corte Constitucional [C.C,] [Constitucional Court], Sala Sexta de Revision, 10 November 2016, M.P.: J. Palacio, Expediente T-5.016.242 (Colom.).

13 Corte Suprema de Justicia [C.S.J.] [Supreme Court], 5 April 2018, STC 4360-2018 (Colom.). Our emphasis.

14 Advisory Opinion OC-23/17 (15 November 2017). Series A No. 23. Solicitada por la República de Colombia, Medio Ambiente y Derechos Humanos.
